# Systematic Analysis of Long Non-Coding RNAs and mRNAs in the Ovaries of Duroc Pigs During Different Follicular Stages Using RNA Sequencing

**DOI:** 10.3390/ijms19061722

**Published:** 2018-06-11

**Authors:** Yi Liu, Mengxun Li, Xinwen Bo, Tao Li, Lipeng Ma, Tenjiao Zhai, Tao Huang

**Affiliations:** 1 College of Animal Science and Technology, Shihezi University, 221 North Fourth Road, Shihezi 832000, China; liuyi_shz@163.com (Y.L.); 18699301319@163.com (M.L.); 15699332887m@sina.cn (T.L.); plmaing2018@sina.com (L.M.); zhai3648@126.com (T.Z.); 2 State Key Laboratory of Sheep Genetic Improvement and Healthy Production, Institute of Animal Husbandry and Veterinary, Xinjiang Academy of Agricultural and Reclamation Sciences, 221 Wu Yi Road, Shihezi 832000, China; xinwen_bo@126.com

**Keywords:** RNA-seq, pig, ovary, lncRNA, mRNA

## Abstract

The dynamic process involving the selection and maturation of follicles is regulated and controlled by a highly synchronized and exquisitely timed cascade of gene expression. Studies have shown that long non-coding RNA (lncRNA) is essential for the normal maintenance of animal reproductive function and has an important regulatory function in ovarian development and hormone secretion. In this study, a total of 2076 lncRNAs (1362 known lncRNAs and 714 new lncRNAs) and 25,491 mRNAs were identified in libraries constructed from Duroc ovaries on days 0, 2 and 4 of follicle development. lncRNAs were shorter, had fewer exons, exhibited a shorter ORF (Open Reading Frame) length and lower expression levels, and were less conserved than mRNAs. Furthermore, 1694 transcripts (140 lncRNAs and 1554 mRNAs) were found to be differentially expressed in pairwise comparisons. A total of 6945 co-localized mRNAs were detected in cis in 2076 lncRNAs. The most enriched GO (Gene Ontology) terms were related to developmental processes. KEGG (Kyoto Encyclopedia of Genes and Genomes) pathway analysis revealed that the differentially expressed lncRNAs targeted mRNAs, and the differentially expressed mRNAs were related to the TGF-β signaling pathway, the PI3K-Akt signaling pathway, the Retinol metabolic pathway and the Wnt signaling pathway. This study deepened our understanding of the genetic basis and molecular mechanisms of follicular development in pigs.

## 1. Introduction

Fecundity is a major factor to be addressed in increasing the efficiency of the pig industry, and litter size is one of the main objective traits for improving pig breeding. Ovulation is the first determinant factor for litter size, and some studies have shown that selection according to ovulation numbers can increase litter size in sows [1]. The ovulation rate of Duroc pigs in each estrous cycle is only 12.3 on average, and because of the decreased follicular phase selection period of this breed, it cannot be treated in the same way as high-yield sows to maintain a larger medium-follicular library [2]. Ovaries are not only important female organs where germ cells mature; but are also important for the organization of hormone secretion. The regulation of ovary and follicle development has recently garnered increased attention. However, the associated mechanisms and genetics underlying this regulation are not yet clear. Each stage in the normal ovarian life cycle involves a highly synchronized and elaborate cascade of gene expression. The regulation of follicular and oocyte maturation is not a simple cascade but rather a complex multifactorial regulatory process that requires specific genes to be accurately expressed at particular times [3].

Long non-coding RNAs (lncRNAs), a class of RNA with lengths greater than 200 nucleotides and no long reading frame, do not encode proteins in eukaryotes [4]. Approximately 5–10% of the genome in a particular cell or tissue will be stably expressed [5], and since only approximately 1% of the genome encodes proteins, approximately 4–9% of the genome consists of non-coding RNAs, of which lncRNAs are the most prevalent [6]. lncRNAs are poorly conserved among species and were previously thought to represent transcriptional noise. However, with further study of lncRNAs, many have been found to be transcriptionally regulated in a tissue-specific or time-specific manner [7], suggesting essential regulatory roles for life activities.

Most reported ovarian lncRNAs are associated with ovarian tumors. The deletion of *H19* can reduce tumor cell invasion and metastasis, and increased expression of *H19* can increase these two characteristics [8]. *XIST* loss leads to Xi (Inactivated X) loss, and thus promotes ovarian tumors [9]. Marsh et al. [10] found that overexpression of the *HOTAIR* gene promotes metastasis of a variety of tumors; in contrast, silencing *HOTAIR* can attenuate ovarian epithelial cell carcinoma, inhibiting ovarian cancer in animal models of peritoneal metastasis. *LSINCT5* is overexpressed in ovarian cancer cell lines and tumor tissues relative to the levels in their normal counterparts. In addition, knocking down the expression of *LSINCT5* in cancer-derived cell lines causes a decrease in cellular proliferation [11].

Pigs are important animals in economic terms and represent a good model animal for biological studies, although only 12,103 pig lncRNAs have been submitted to any database (ALDB) [12]. In contrast, there are 198,619 lncRNAs available for humans (Release 23) and 111,706 for mice (Release M7) in the Gencode database. Previous studies have identified a limited number of porcine lncRNAs using RNA-seq. For example, 6621 pig lncRNA transcripts corresponding to 4515 lncRNA gene loci were derived from 93 samples [13]; 570 lncRNA transcripts from 476 lncRNA gene loci were identified from fetal porcine skeletal muscle [14]; 777 lncRNA transcripts from 752 lncRNA gene loci were identified from porcine testes [15]; and 2805 lncRNA putative non-coding transcripts were obtained from twenty Yorkshire gilts [16]. However, no porcine ovarian lncRNAs have been reported.

In this study, RNA-seq was used to investigate the expression profiles of ovarian tissues on days 0 (DOVADAY0), 2 (DOVADAY2) and 4 (DOVADAY4) of the follicular phase. We aimed to determine the types and numbers of transcripts (mRNA and lncRNA) that are differentially expressed in the ovaries at different stages and how they relate to ovarian development to provide new insight into the molecular mechanism and regulatory network of porcine follicular development and reproductive-trait-related lncRNAs.

## 2. Results

### 2.1. Data Output Summary of RNA Sequencing Identification

A total of 89,575,480, 92,141,736 and 111,985,046 raw reads were generated for DOVADAY0, DOVADAY2 and DOVADAY4, respectively. The GC contents of each library were 54.21%, 54.04% and 53.06%. In addition, 86,494,304 (96.56%), 88,644,926 (96.20%) and 109,301,566 (97.60%) clean reads were retained and used in following analysis after discarding reads with adapters or a poly-N content >10% and other low-quality reads. Approximately 80.14%, 81.31% and 82.82% of the clean reads from each library were mapped to the pig reference genome (*Sus scrofa* 10.2), and 96,720 transcripts were assembled in these three libraries using Scripture and Cufflinks.

### 2.2. Identification of mRNAs and lncRNAs in the Ovary

**Figure 1:**
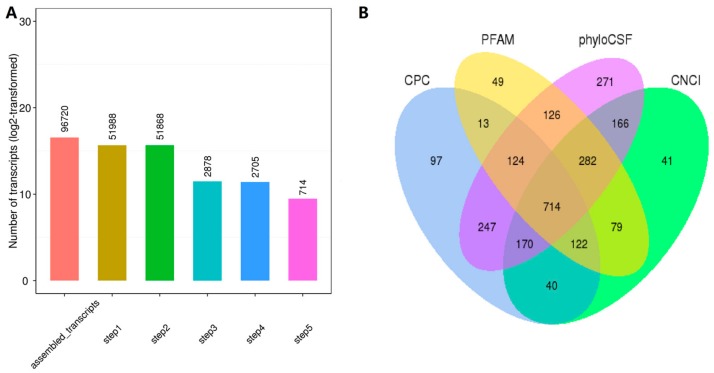
Screening of the candidate lncRNAs in the ovarian transcriptome. (**A**) 96,720 transcripts were assembled by using Cufflinks and Scripture with a stringent filtering pipeline to discard transcripts without all characteristics of lncRNA (see the methods for specific steps); (**B**) Identification of non-coding lncRNAs by using CPC, PFAM, phyloCSF and CNCI. Seven hundred and fourteen non-coding transcripts were identified by the four software programs, and both protein-coding transcripts and putative protein-coding transcripts were removed (step 5 above).

To minimize the false positive rate in the identification of lncRNAs from the 96,720 assembled transcripts, we developed a stringent filtering pipeline to discard transcripts lacking any lncRNA characteristics (Figure 1A). After discarding transcripts with a length of less than 200 bp, fewer than two exons or an FPKM (Fragments Per Kilobase of exon model per Million mapped reads) value of less than 0.5, the remaining transcripts were subjected to Blast searches against known pig lncRNAs, removing known classes of RNA (tRNA, rRNA, snRNA, snoRNA, pre-miRNA, and pseudogenes) and protein-coding genes. A total of 1362 known pig lncRNAs were recovered. As the identification of transcripts included immature mRNA fragments, we used four tools (CPC, CNCI, pfam and phyloCSF) to remove potential coding transcripts. Finally, 714 putative non-coding transcripts were retained, which included 626 lincRNAs (87.70%), 88 anti-sense lncRNAs (12.3%) and 0 intronic lncRNAs (0%) (Figure 1B). A total of 2076 lncRNAs (1362 annotated lncRNAs and 714 novel lncRNAs, Table S1) were used in subsequent analyses. In addition, 25,491 mRNAs were identified.

### 2.3. Comparison of lncRNA and mRNA Characteristics

**Figure 2:**
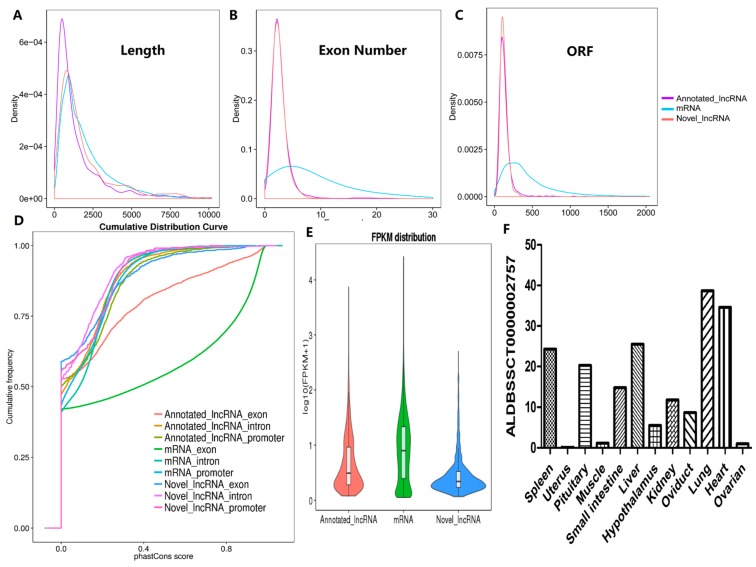
Comparison of candidate lncRNAs and mRNAs characteristics. (**A**) Length, (**B**) exon number, (**C**) open reading frame (ORF) length distribution of mRNAs, annotated-lncRNAs and novel-lncRNAs. (**D**) Conservation score for mRNAs, annotated-lncRNAs and novel-lncRNAs by using phasCon software. (**E**) A violin plot of expression level (showed in log10 (FPKM + 1)) for mRNAs, annotated-lncRNAs and novel-lncRNAs. (**F**) The expression of ALDBSSCT0000002757 in twelve tissues.

In this study, we described the characteristics of 2076 lncRNAs and 25,491 mRNAs. Our results indicated that the previously known and novel lncRNAs were shorter than mRNAs (Figure 2A); their expressions levels were lower than those of mRNAs (Figure 2E); and their genes tended to contain fewer exons (Figure 2B). We also found that our lncRNAs were less conserved than protein coding transcripts through a phastCon analysis (Figure 2D). Interestingly, conservative comparison of mRNA 5′UTR, 3′UTR and CDS regions exons showed the same high conservative in contrast with the exons of lncRNAs (Figure S1. Furthermore, the identified lncRNAs tended to have shorter ORFs than mRNAs (Figure 2C). Our results were observed to be consistent with previous studies [13,14,15,16]. The accuracy of the selected lncRNAs was further confirmed by comparative analysis of structural features.

Another characteristic of lncRNAs is that they exhibit obvious temporal and spatial expression specificity during tissue differentiation and development. We next examined the expression of nine selected lncRNAs in 12 tissues (Figures S2 and Figure 2F). Total RNA was extracted from the heart, liver, spleen, lung, kidney, small intestine, hypothalamus, pituitary, uterus, ovary, muscle and fallopian tubes. The expression of ALDBSSCT0000002757 in these tissues was detected via qRT-PCR, which showed that the expression of ALDBSSCT0000002757 presented obvious tissue specificity (ENSSSCT and ALDBSSCT were known lncRNAs from the ENSEMBL and ALDB databases, and the new selected lncRNAs were designated LNC).

### 2.4. qRT-PCR Confirmation and Differential Expression Analysis

**Figure 3:**
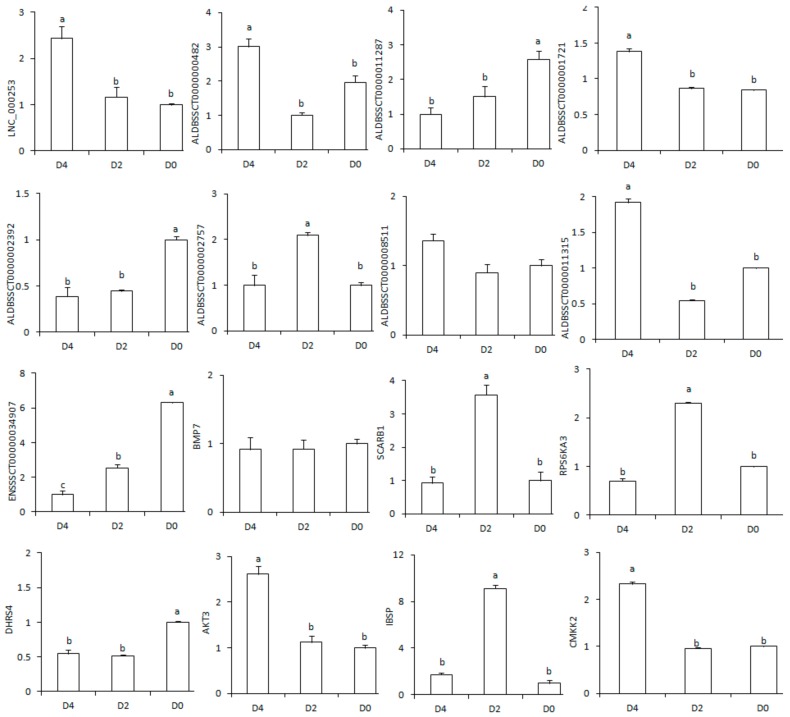
Confirmation of the expression patterns of lncRNA and mRNA using qRT-PCR. Nine random differentially expressed lncRNAs and seven mRNAs from the three compared groups were analyzed by qRT-PCR. Transcript expression was quantified relative to the expression level of *GAPDH* using the comparative cycle threshold (−ΔΔ*C*_t_) method. The column height represents the fold change compared with the control sample. Different superscript letters between columns indicate a significant difference (*p* < 0.05).

The expression levels of the genes calculated by Cuffdiff should be verified prior to analysis of differences. DEG analysis and qPCR experiments were all performed at the isoform level. To verify the RNA-seq results, nine lncRNAs were selected from the three libraries (ENSSSCT00000034907, ALDBSSCT0000002757, ALDBSSCT0000002392, ALDBSSCT0000001721, ALDBSSCT0000011315, LNC_000253, ALDBSSCT0000000482, ALDBSSCT0000011287, and ALDBSSCT0000008511) for qRT-PCR. The selected lncRNAs were significantly different in at least one sample comparison and exhibited predicted target genes that have been reported to be involved in ovarian development. The results showed that, with the exception of ALDBSSCT0000008511, the expression patterns of these genes were consistent with the RNA-seq results. The changes in ALDBSSCT0000008511 in the ovary at different developmental stages were not obvious, possibly due to the low expression of this lncRNA. To verify the accuracy of the mRNA-related data, seven mRNAs were selected. These target genes were involved in ovarian development-related pathways. *IBSP* is involved in the PI3K-Akt signaling pathway, *AKT3* in the PI3K-Akt and FOXO signaling pathways, *BMP7* in the TGF-β signaling pathway, and *CAMKK2* in the oxytocin signaling pathway, while *RPS6KA3* is important for progesterone-mediated oocyte maturation and oocyte meiosis, *SCARB1* for ovarian steroid production, and *DHRS4* for retinol metabolism. These results were consistent with the results of high-throughput sequencing (Figure 3).

Using |log2(fold change)| > 1 and *p*-adjusted < 0.05 as cutoffs, 1694 (140 lncRNAs and 1554 mRNAs) differentially expressed genes (DEGs) were obtained from pairwise comparisons of samples collected from Duroc pigs on days 0, 2 and 4 of the follicular period (DOVADAY2 vs. DOVADAY0; DOVADAY4 vs. DOVADAY0; and DOVADAY4 vs. DOVADAY2, Figure S3). Thirty-nine lncRNA transcripts (8 up-regulated and 31 down-regulated) and 521 mRNA transcripts (220 up-regulated and 301 down-regulated) were differentially expressed between DOVADAY0 and DOVADAY2 (Table S2). Fifty-three lncRNA transcripts (37 up-regulated and 16 down-regulated) and 603 mRNA transcripts (432 up-regulated and 171 down-regulated) were differentially expressed between DOVADAY2 and DOVADAY4 (Table S3). Forty-eight lncRNA transcripts (30 up-regulated and 18 down-regulated) and 430 mRNA transcripts (260 up-regulated and 170 down-regulated) were differentially expressed between DOVADAY4 and DOVADAY0 (Table S4). As shown in Table 1, 17 DEGs were common to the three comparisons (1 lncRNA and 16 mRNAs). None of the obtained lncRNAs were previously identified. The differentially expressed lncRNAs and mRNAs were widely distributed across almost every chromosome. In an unsupervised hierarchical clustering analysis, heat maps were generated using the differentially expressed lncRNAs and mRNAs, which clearly self-segregated into DOVADAY0, DOVADAY2 and DOVADAY4 (Figure S4).

### 2.5. Differentially Expressed lncRNAs Target Genes Prediction and GO and KEGG Enrichment Analyses

Most of the known lncRNAs are not functionally annotated in current databases. Previous studies have demonstrated that lncRNAs act in cis and regulate the expression of nearby protein-coding genes [17]. To evaluate the potential cis regulatory functions of lncRNAs, we searched for protein-coding genes 100 kb upstream and downstream of the differentially expressed lncRNAs. A total of 6945 co-localized mRNAs were detected in cis between the 2076 lncRNAs and the protein-coding genes in the pig genome (Table S5). Interestingly, we detected genes related to reproductive traits, such as *ESR1*, *FSHR*, *ITGB1*, *BMPR2*, *BMPR1A* and *BMP15*, which were located near the ALDBSSCT0000000657, LNC_000494, ALDBSSCT0000001293, ALDBSSCT0000004580, ALDBSSCT0000004000 and ENSSSCT00000035247 loci, respectively (Table 2).

We then performed GO and KEGG pathway enrichment analyses of the co-localized mRNAs to predict the functions of the differentially expressed lncRNAs, but no significant GO enrichment results were obtained, since the number of co-localized mRNAs was relatively small. KEGG pathway analysis showed that several pathways were related to ovarian development, for example, the Wnt signaling pathway, the PI3K-Akt signaling pathway, ovarian steroidogenesis, retinol metabolism, the oxytocin signaling pathway, the estrogen signaling pathway, the GnRH signaling pathway, the TGF-β signaling pathway, the FoxO signaling pathway, progesterone-mediated oocyte maturation, oocyte meiosis and the prolactin signaling pathway. These findings suggested that lncRNAs act on their neighboring protein-coding genes in cis to regulate ovarian development.

### 2.6. GO and KEGG Enrichment Analyses of Differentially Expressed mRNAs

**Figure 4:**
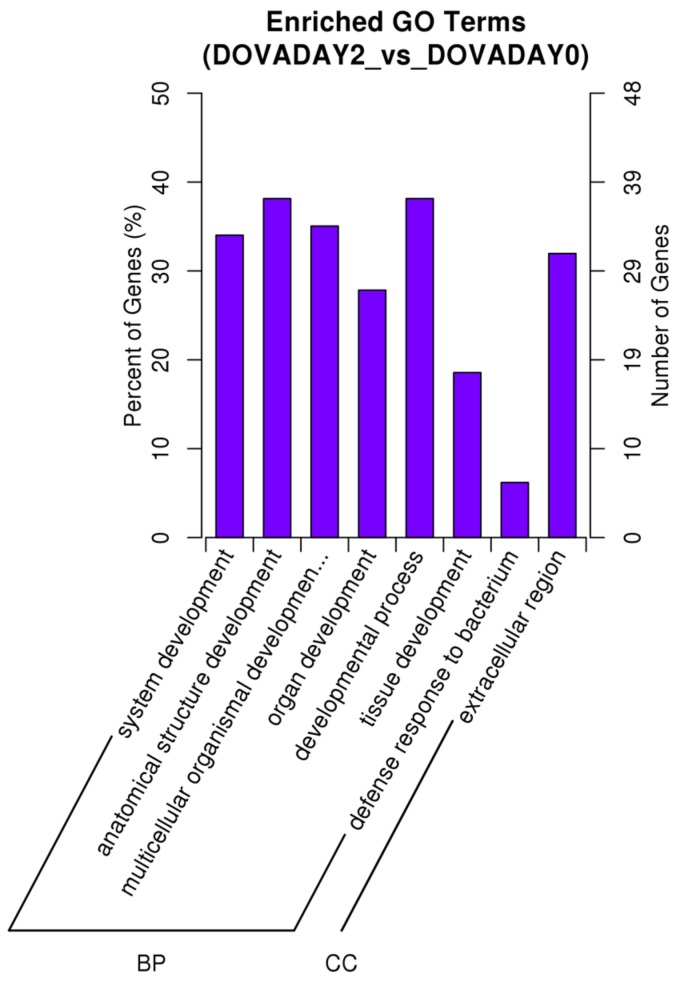
GO enrichment plot of differentially expressed mRNAs between DOVADAY2 and DOVADAY0. BP: biological process; CC: cell composition; MF: Molecular function.

The predicted functional categories of the dysregulated lncRNAs were closely related to those of the dysregulated protein-coding genes, indicating a potential strong connection and interaction between lncRNAs and mRNAs. To ascertain the functions of the differentially expressed mRNAs and the connections among them, we performed GO term and KEGG pathway enrichment analyses. For the GO analysis of the differentially expressed mRNAs between DOVADAY2 and DOVADAY0, the significantly over-represented terms are shown in Figure 4, including the following biological process terms: System development, anatomical structure development, multicellular organismal development, organ development, developmental process, tissue development and defense response to bacterium. Notably, the only cellular component term was extracellular region. No significant GO enrichment results were found for the other two comparisons (DOVADAY4 vs. DOVADAY0 and DOVADAY4 vs. DOVADAY2).

The KEGG pathway analyses mapped the dysregulated protein-coding genes to KEGG reference pathways to infer systemic biological behaviors. The KEGG pathway analysis of the differentially expressed mRNAs between DOVADAY2 and DOVADAY0 revealed that starch and sucrose metabolism, steroid hormone biosynthesis, rheumatoid arthritis, leishmaniasis, bile secretion, carbohydrate digestion and absorption, cell adhesion molecules (CAMs), ECM-receptor interaction, pentose and glucuronate interconversion and ABC transporters were significantly over-represented (Figure S5). In the comparison of DOVADAY4 and DOVADAY0, starch and sucrose metabolism, ECM-receptor interaction, drug metabolism—cytochrome P450, retinol metabolism, carbohydrate digestion and absorption, steroid hormone biosynthesis and ABC transporters were significantly over-represented (Figure S6). Finally, in the comparison of DOVADAY2 and DOVADAY4, ECM-receptor interaction, focal adhesion, phagosome, leishmaniasis, the PI3K-Akt signaling pathway, rheumatoid arthritis and ABC transporters were significantly over-represented (Figure S7). Although the same over-represented terms appeared in multiple comparisons, the genes enriched in the terms were distinct among the compared samples. For example, ECM-receptor interaction and ABC transporters were over-represented in all three compared samples, but retinol metabolism was specifically highly expressed on DOVADAY4, while the PI3K-Akt signaling pathway was specifically highly expressed on DOVADAY2.

## 3. Discussion

The development of follicles involves the processes of recruitment, selection and dominance [18]. Normally, the porcine follicle recruitment process occurs on the 14th day of periodic development or weaning, and the selection process occurs during the estrus cycle at 16–20 days. On the 16th day, there are 40–50 follicles that are 2–6 mm in diameter in the ovaries of the sows, among which only a small number of follicles are selected for maturation to ovulation, and most of the follicles undergo follicular atresia for the regulation of apoptosis. The number of follicles discharged depends on the early follicle recruitment and selection processes. Therefore, the 14th–18th days of estrus (0–4th days of the follicular period) were chosen in this experiment to study the physiological regulation of follicular recruitment and selection.

Our newly identified pig ovarian lncRNAs shared many characteristics with those of other mammalian species. They were shorter and exhibited fewer exons, shorter ORFs, and lower expression levels and conservation rates than protein-coding transcripts. We also found that our lncRNAs were less conserved than lncRNAs found in human [19] and mouse [7] tissues. We also observed that the ovarian lncRNAs were shorter than those present in endometrial tissues [16] (1454 bp on average) but showed a similar number of exons. Interestingly, the ovarian pig lncRNAs were shorter than the lncRNAs of humans (1644 bp on average) and goats [20] (1809 bp on average), but longer than those of mice (550 nt on average) and contained fewer exons than mouse lncRNAs (3.7 exons on average).

Most of the available evidence suggests that the expression of lncRNAs can regulate and show high correlations with the expression of neighboring mRNAs [21]. Interestingly, we detected reproductive trait-related genes such as *ESR1*, *FSHR*, *ITGB1*, *BMPR2*, *BMPR1A* and *BMP15*, which were located near ALDBSSCT0000000657, LNC_000494, ALDBSSCT0000001293, ALDBSSCT0000004580, ALDBSSCT0000004000 and ENSSSCT00000035247, respectively. Follicular maturation processes require estrogen, follicle-stimulating hormone (FSH) and luteinizing hormone (LH) stimulation. Ovaries mainly secrete estrogen and progesterone, and ovarian granulosa cells are an important location for estrogen synthesis. Ovarian endometrial cells transform cholesterol into androstenedione under the influence of LH, and ovarian granulosa cells produce an enzyme that promotes the transition of androstenedione into estrogen in the developmental process, after which the estrogen is secreted into the follicular fluid and blood. FSH plays an important role in promoting follicular growth (including granulocyte cell proliferation, endometrial cell differentiation and follicular fluid secretion) and maturation processes. FSH requires the synergistic effect of LH to promote follicular maturation ovulation. Many researchers have studied the relationship between estrogen receptor gene (ESR) gene polymorphism and total litter size [22]. Most studies have shown that *ESR* is polymorphic in various pig breeds and has a significant effect on the total litter size of pigs. Although there have been some reported differences, most results have indicated that the B allele of *ESR* is able to increase the total litter size and exhibits a BB > AA effect. The *ESR* and follicle stimulating hormone beta subunit gene (*FSHβ*) have been shown to be the two major genes affecting swine litter size [23].

Studies have confirmed that TGF-β family members participate in the regulation of ovarian function and ovarian follicular development [24]. Approximately 5% of primordial follicles in each cycle are activated and develop to the growing follicle stage and, ultimately, into a luminal follicle that can drain mature oocytes after the establishment of primitive follicles in female mammals (mice, pigs, humans). It has been shown that the TGF-β signaling pathway can regulate the maintenance and activation of primordial follicles in the mouse ovary by regulating the PI3K-Akt signaling pathway. At the same time, PI3K-Akt can effectively activate the Wnt signaling pathway [25], and the Wnt signaling pathway is involved in the apoptosis of porcine ovarian granulosa cells. KEGG enrichment analysis revealed that a large number of target mRNAs of differentially expressed lncRNAs and differentially expressed mRNAs were associated with the TGF-β, PI3K-Akt and Wnt signaling pathways. For example, ALDBSSCG0000006872 and XLOC_065146 target the mRNA of *BMP7*, which belongs to the TGF-β family, and it has been reported that several days after the injection of BMP7 in 28-day-old rats, the rat ovary exhibits more primary follicles and preantral follicles and a follicular cavity [26]. *BMP7* promotes the proliferation of small granulosa cells in vitro, suggesting that *BMP7* may promote primordial follicle development by fostering the development of follicles into preantral follicles, which may contribute to primordial follicle recruitment by promoting the development of primary follicles [27]. *ITGB1* is the target mRNA of ALDBSSCG0000007347, XLOC_058285, XLOC_065146, XLOC_049208 and ALDBSSCG0000007201. *ITGB1* is a member of the integrin family and the PI3K-Akt signaling pathway. Early laboratory experiments showed that the TT, TC and CC genotypes were amplified from the *ITGB1* gene in large white pigs. Correlation analysis revealed that the total litter size of pigs carrying the CC genotype was larger than that of pigs with the TT genotype. The *ITGB1* gene may be associated with the reproductive performance of sows. Hoogeboom et al. [28] showed that the Wnt signaling pathway is involved in follicular development through negative regulation, which affects granulosa cells and follicular steroid synthesis through activation of the transcription factor *Foxo3*. However, RNA-Seq results showed that *Foxo3* presented no significant difference in the three comparisons, whereas the level of *DKK2* (dickkopf inhibitor 2) was significantly higher on DOVADAY0. *DKK2* is a key suppressor of the Wnt signaling pathway. The expression of *DKK2* gradually decreased during the selection and recruitment of follicles, indicating that this process was accompanied by apoptosis of follicles.

ENSSSCT00000034907, the target gene of which is *DHRS4* (short-chain fatty alcohol dehydrogenase), was the only lncRNA showing significant differential expression in the three pools. *DHRS4* is involved in the retinol metabolic pathway and is responsible for synthesizing *NRDR* (coenzyme II-dependent retinol dehydrogenase), a key enzyme in the synthesis of retinoic acid. *DHRS4* and its downstream homologous genes *DHRS4L1* and *DHRS4L2* constitute the *DHRS4* gene cluster and are arranged in tandem on human chromosome 14 at site 14q11.2. The antisense lncRNA *AS1DHRS4* affects this cluster through cis and trans effects, altering its expression by regulating methylation status and modifying chromosome conformation [29]. In this study, qRT-PCR experiments showed that the expression of ENSSSCT00000034907 and *DHRS4* was much higher on DOVADAY0 than DOVADAY2 and DOVADAY4. Preliminary results indicated that *RBP4* (retinol-binding protein), which participates in the retinol metabolic pathway, is a candidate gene for litter size because of its involvement in the early development of the embryo. Furthermore, in the pig uterine cavity, the nourishing ectoderm can secrete *RBP4* [30]. The regulation of *DHRS4* expression by ENSSSCT00000034907 and their link to *RBP4* require further study.

In conclusion, we identified lncRNA and mRNA expression profiles for pig ovaries on days 0, 2, and 4 of the follicular period in Duroc pigs. Importantly, we analyzed the genomic features, differential expression, and target pathways of all of the identified lncRNAs. As the roles of lncRNAs in pigs have not yet been fully elucidated, these findings provide a valuable resource for research on both ovarian development and ovarian-follicle-related genes in pigs and may also provide clues for understanding similar molecular mechanisms in other mammalian species. In addition, our ongoing efforts will focus on the functions of these lncRNAs through experimental approaches, which should provide a richer understanding of the genetic basis and molecular mechanisms of high prolificacy in pigs.

## 4. Materials and Methods

### 4.1. Ethics Statement and Sample Collection

All experiments involving animals were conducted under a protocol approved by the Medical Ethics Committee, First Affiliated Hospital, Medical College, Shihezi University (2014-073-01, 5 March 2014). Eight Duroc gilts with similar ages and genetic backgrounds were selected from the Xinjiang Tiankang Animal Husbandry Biotechnology Co., Ltd. The animals were allowed access to feed and water until the appearance of estrus and were injected with veterinary chloroprostenol on day 14. Ovaries were collected from animals slaughtered 48h after injection, which was recorded as DOVADAY2 (*n* = 3), and from animals slaughtered 96h after injection, which was recorded as DOVADAY4 (*n* = 3). The ovaries of the other two animals were collected on the day of injection, which was recorded as DOVADAY0 (*n* = 2). Each ovary was flushed with PBS (pH 7.4), and the tissue samples were frozen in liquid nitrogen and stored at −80 °C until RNA isolation.

### 4.2. Total RNA Isolation and Qualification

Total RNA from each sample was isolated using the TRIzol reagent (Invitrogen, Carlsbad, CA, USA). Agarose gel electrophoresis was performed to assess the degradation and contamination of RNA. The purity and concentration of the total RNA were checked using a NanoPhotometer spectrophotometer (Implen, Los Angeles, CA, USA) and a Qubit RNA Assay Kit, with a Qubit 2.0 Fluorometer (Life Technologies, Carlsbad, CA, USA). In addition, RNA integrity was assessed using an RNA Nano 6000 Assay Kit on a Bioanalyzer 2100 (Agilent Technologies, Santa Clara, CA, USA).

### 4.3. Library Preparation for lncRNA Sequencing

In this study, three cDNA libraries, designated DOVADAY0, DOVADAY2 and DOVADAY4, were constructed using the NEBNext Ultra Directional RNA Library Prep Kit for Illumina (NEB, Ipswich, MA, USA), after the removal of ribosomal RNA with the Epicentre Ribo-zero rRNA Removal Kit (Epicentre, Madison, WI, USA). Briefly, rRNA-depleted RNA was fragmented using NEBNext First Strand Synthesis Reaction Buffer (5X). Then, the first strand of cDNA was synthesized using random hexamer primers and M-MuLV Reverse Transcriptase (RNase H-), and the synthesis of the second strand cDNA was performed using DNA Polymerase I, RNase H and reaction buffer. In the reaction buffer, dUTP replaced dTTP in the dNTP mixture. To select cDNA fragments of the preferred length of 150–200 bp, library fragments were purified with an AMPure XP system (Beckman Coulter, Brea, CA, USA). The purified double-stranded cDNAs were further repaired via A-tailing and ligated to sequencing adapters. Then, 3 μL of USER Enzyme (NEB, Ipswich, MA, USA) was combined with size-selected, adaptor-ligated cDNA at 37 °C for 15 min, followed by 5 min at 95 °C before PCR. Subsequent PCR was performed with Phusion High-Fidelity DNA polymerase, universal PCR primers, and Index (X) Primers. Finally, the products were purified with the AMPure XP System, and an Agilent Bioanalyzer 2100 was used to assess library quality. After clustering the index-coded samples using a cBot Cluster Generation System with the TruSeq PE Cluster Kit v3-cBot-HS (Illumina), the libraries were sequenced at the Novogene Bioinformatics Institute (Beijing, China) on the Illumina HiSeq 2500 platform, generating 125 bp paired-end reads.

### 4.4. Quality Analysis, Mapping, and Transcriptome Assembly

Clean reads were obtained by removing low-quality reads and those containing adapters or poly-N sequences from the raw data using in-house Perl scripts. At the same time, Q20, Q30 and GC content values were calculated for the clean data. All subsequent analyses were based on the high-quality data. The reference genome and gene model annotation files were downloaded from the Ensembl genome browser (*Sus scrofa* 10.2, http://www.ensembl.org/index.html). An index of the pig reference genome was built using Bowtie v2.0.6 [31], and clean paired-end reads were aligned to the reference genome using TopHat v2.0.9 [32]. The mapped reads from each sample were assembled using both Scripture (beta2) [33] and Cufflinks v2.1.1 [34].

### 4.5. Expression and Conservative Analysis

Cuffdiff (v2.1.1) was used to calculate fragments per kb per million reads (FPKM) values for both the lncRNAs and coding genes in each sample. Transcripts showing an adjusted *p* < 0.05 and |log2(fold change)| > 1 among DOVADAY0, DOVADAY2 and DOVADAY4 were described as differentially expressed. To investigate the sequence conservation of the transcripts, we employed two important statistical programs in the Phast (v1.3) package: phyloFit and phastCons. PhyloFit was used to compute phylogenetic models for conserved and non-conserved regions among species and to then construct a model and HMM transition parameters for phastCons, to compute the conservation scores of the lncRNAs and coding genes [35].

### 4.6. lncRNAs Identification

To reduce the rate of false positives, we employed the following five steps to identify lncRNAs, including lincRNAs, intronic lncRNAs and anti-sense lncRNAs [21]. All of the assembled transcripts from two sequencing libraries were combined using the software cuffcompare [36], and transcripts were discarded if they were only assembled by either Scripture (beta2) or Cufflinks v2.1.1. We set up a series of strict screening conditions according to the lncRNA characteristics based on the results of the combined Cufflinks and Scripture assemblies, resulting in the selection of a final set of lncRNA candidates for subsequent lncRNA analysis. (1) Transcripts were removed if they contained fewer than two exons; (2) transcripts < 200 bp in length were also discarded; and (3) the remaining transcripts were subjected to Blast searches with known pig lncRNAs in ALDB [12] using Cuffcompare. Only lncRNA transcripts with completely congruent splice sites between our results and those of ALDB were immediately categorized as known lncRNAs. In addition, transcripts that were determined to be rRNAs, tRNAs, snRNAs, snoRNAs, pre-miRNAs or pseudogenes were discarded, and (4) the expression of each transcript was calculated using Cuffquant. Transcripts with an FPKM ≥ 0.5 were selected. Finally, (5) four software packages for coding potential analysis were used to identify candidate lncRNAs: Coding-Non-Coding–Index (CNCI) (score < 0) [37], Coding Potential Calculator (CPC) (score < 0) [38], Pfam-scan (E-value < 0.001) [39] and Phylogenetic Codon Substitution Frequency (phyloCSF) (Max_score ≤ 100) [40], and the intersections of the results from each program were defined as the collection of novel lncRNA transcripts. Then, we selected those shared across the four tools as the final candidate set of lncRNAs and employed these lncRNAs for further analysis.

### 4.7. Target Gene Prediction and GO and KEGG Enrichment Analyses

To explore lncRNA functions, we first predicted the target genes of the lncRNAs in cis and trans. A cis role for a lncRNA indicates that it acts on neighboring target genes. Briefly, the coding genes 100 K upstream and downstream of a lncRNA were searched for evidence of cis roles. A trans role refers to the identification of other genes based on correlated expression levels, and results with Pearson’s correlation coefficients (|r| > 0.95) were selected. To understand the functional roles of the target genes of the lncRNAs, we used the GOseq R package to implement enrichment analysis. In addition, the differentially expressed protein-coding genes were also analyzed using GO. KEGG is a database resource for understanding high-level functions and utilities of biological systems. Therefore, we employed the KOBAS software to detect the enrichment of lncRNA target genes or differentially expressed genes in KEGG pathways [41]. GO terms and KEGG pathways with a corrected *p* < 0.05 were considered significantly enriched.

### 4.8. qRT-PCR Verification

The results of RNA sequencing were validated by qRT-PCR. Total cDNA was synthesized using a reverse transcriptase kit (TaKaRa, Dalian, China). qRT-PCR was performed with a SYBR green assay (TaKaRa Biotechnology, Dalian, China) on a Roche LightCycler 480 (Roche Applied Science, Mannheim, Germany). The specific quantitative primers for 16 transcripts are listed in Table S6. Each 25 µL reaction volume contained 8.5 µL H2O, 1 µL of each primer, 2 µL of cDNA, and 12.5 µL of 2 × Real Master Mix (TaKaRa Biotechnology). The conditions were as follows: An initial single cycle (95 °C for 3 min), followed by 40 cycles (95 °C for 15 s; the optimized annealing temperature for 15 s; 72 °C for 20 s) and a final extension step at 72 °C for 5 min. In addition, 10 μL of each PCR product was separated via 2% agarose gel electrophoresis. All amplifications were followed by dissociation curve analysis of the amplified products. Specific primers were designed using Primer Premier 5.0 and Oligo7.37, and their specificities were confirmed using BLAST. Gene expression levels were normalized to *GAPDH* to determine relative expression using the 2 (−ΔΔCt) value method. Significant differences in gene expression were analyzed by SAS9.1 during the various estrous ovarian development stages.

## Figures and Tables

**Table 1:** Seventeen differentially expressed transcripts common to all three samples.

Transcript_ID	Official_Symbol	Gene_Description
ENSSSCT00000034907	*TMP-CH242-74M17.6*	
ENSSSCT00000014468	*LRP4*	LDL receptor related protein 4
ENSSSCT00000033666	*SSR4*	signal sequence receptor, delta
ENSSSCT00000001002	*EPYC*	epiphycan
ENSSSCT00000000530	*LYZ*	Lysozyme C-2
ENSSSCT00000034512	*IBSP*	integrin-binding sialoprotein
ENSSSCT00000018997		
ENSSSCT00000005448	*SLC24A1*	solute carrier family 24 (sodium/potassium/calcium exchanger), member 1
ENSSSCT00000005998	*TXN*	*Sus scrofa* thioredoxin (*TXN*), mRNA.
ENSSSCT00000011983	*CTSL*	cathepsin L
ENSSSCT00000025632	*FAM101A*	family with sequence similarity 101 memberA
ENSSSCT00000012333	*XIRP1*	xin actin binding repeat containing 1
ENSSSCT00000017800		
ENSSSCT00000011289		
ENSSSCT00000024662	*SCG2*	*Sus scrofa* secretogranin II (*SCG2*), mRNA.
ENSSSCT00000006577	*SNTB1*	syntrophin, beta 1 (dystrophin-associatedprotein A1, 59 kDa, basic component 1)
ENSSSCT00000033082	*CHTOP*	chromatin target of *PRMT1*

The first transcript is a lncRNA, the rest are all mRNAs.

**Table 2:** Genomic association between lncRNAs and nearby reproductive-trait-related genes.

lncRNA_ID	lncRNA_Status	mRNA	Distance	Location
ALDBSSCT0000000657	Annotated_lncRNA	*ESR1*	37,317	upstream
ENSSSCT00000035465	Annotated_lncRNA	*GNRHR2*	8321	upstream
LNC_000161	Novel_lncRNA	*SMAD2*	26,321	downstream
LNC_000010	Novel_lncRNA	*SMAD1*	1297	downstream
LNC_000494	Novel_lncRNA	*FSHR*	78,273	downstream
ALDBSSCT0000006453	Annotated_lncRNA	*GDF9*	97,300	downstream
ALDBSSCT0000007736	Annotated_lncRNA	*TGFBR3*	72,194	upstream
LNC_000464	Novel_lncRNA	*TGFBI*	−1125	antisense
ALDBSSCT0000007667	Annotated_lncRNA	*GNRHR2*	23,672	upstream
LNC_000248	Novel_lncRNA	*TGFBR2*	69,500	upstream
ALDBSSCT0000001293	Annotated_lncRNA	*ITGB1*	36,274	downstream
ENSSSCT00000035247	Annotated_lncRNA	*BMP15*	69,930	upstream

## Supplementary Materials

Supplementary materials can be found at http://www.mdpi.com/1422-0067/19/6/1722/s1.

## Funidng

This work was supported by the National Natural Science Foundation of China (31460586).
