# Supplementary material for: Systematic Analysis of Long Non-Coding RNAs and mRNAs in the Ovaries of Duroc Pigs During Different Follicular Stages Using RNA Sequencing
Source: Int J Mol Sci. 2018 Jun 11;19(6):1722. doi: 10.3390/ijms19061722 (PMC6032137; doi:10.3390/ijms19061722)
Supplement: Supplementary file 1 [file ijms-19-01722-s001.zip › Supplementary Figure.pdf]

Systematic analysis of long non-coding RNAs and mRNAs in the ovaries of Duroc pigs during different follicular stages using RNA sequencing

Yi Liu, Mengxun Li, Xinwen Bo, Li Tao, Lipeng Ma, Tenjiao Zhai, Tao Huang

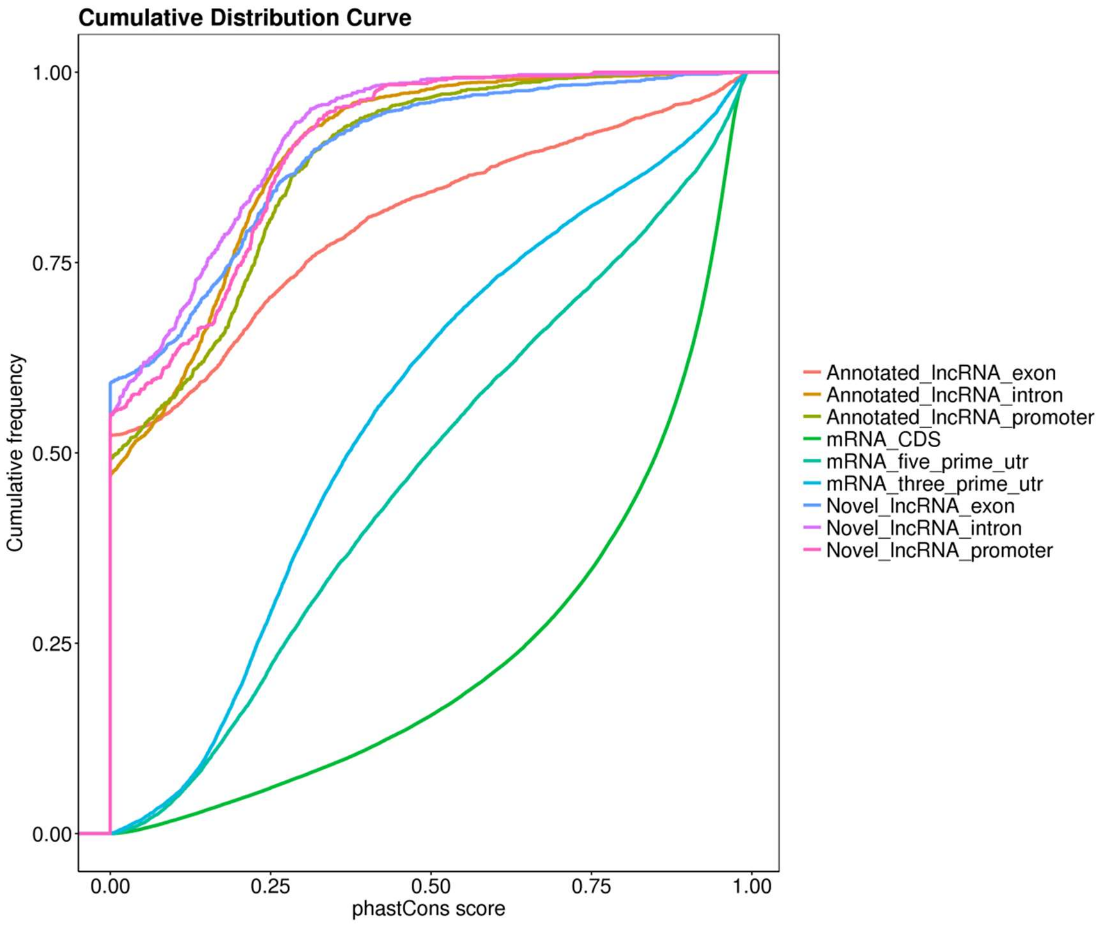

Supplementary Figure 1. Conservation score for mRNAs, annotated-lncRNAs and novel-lncRNAs by using phasCon software.

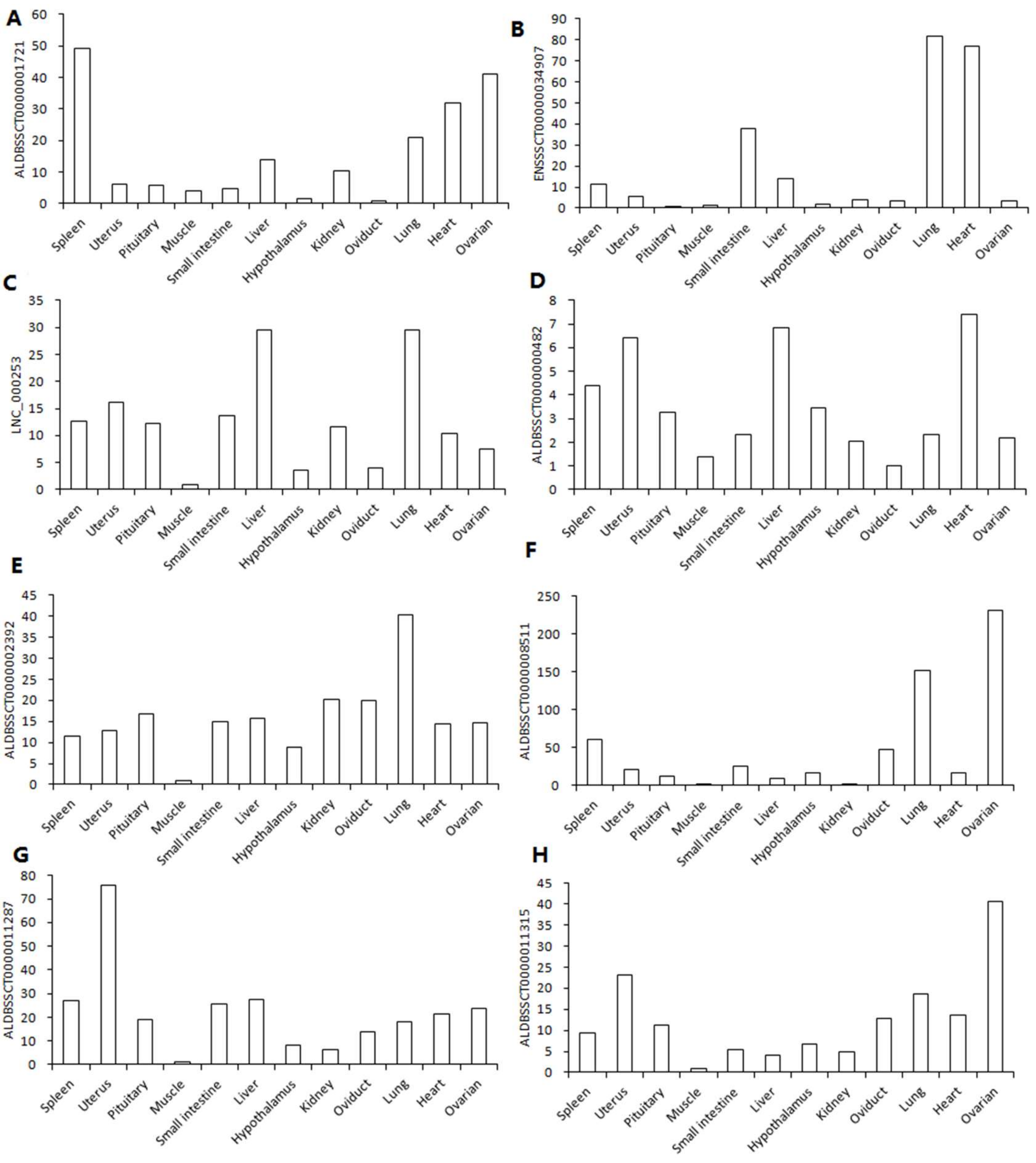

Supplementary Figure 2. The expression of ALDBSSCT0000001721 (A) , ENSSSCT00000034907 (B) , LNC_000253 (C), ALDBSSCT0000000482(D), ALDBSSCT0000002392(E), ALDBSSCT0000008511(F), ALDBSSCT0000011287(G) and ALDBSSCT0000011315(H) in porcine twelve tissues.

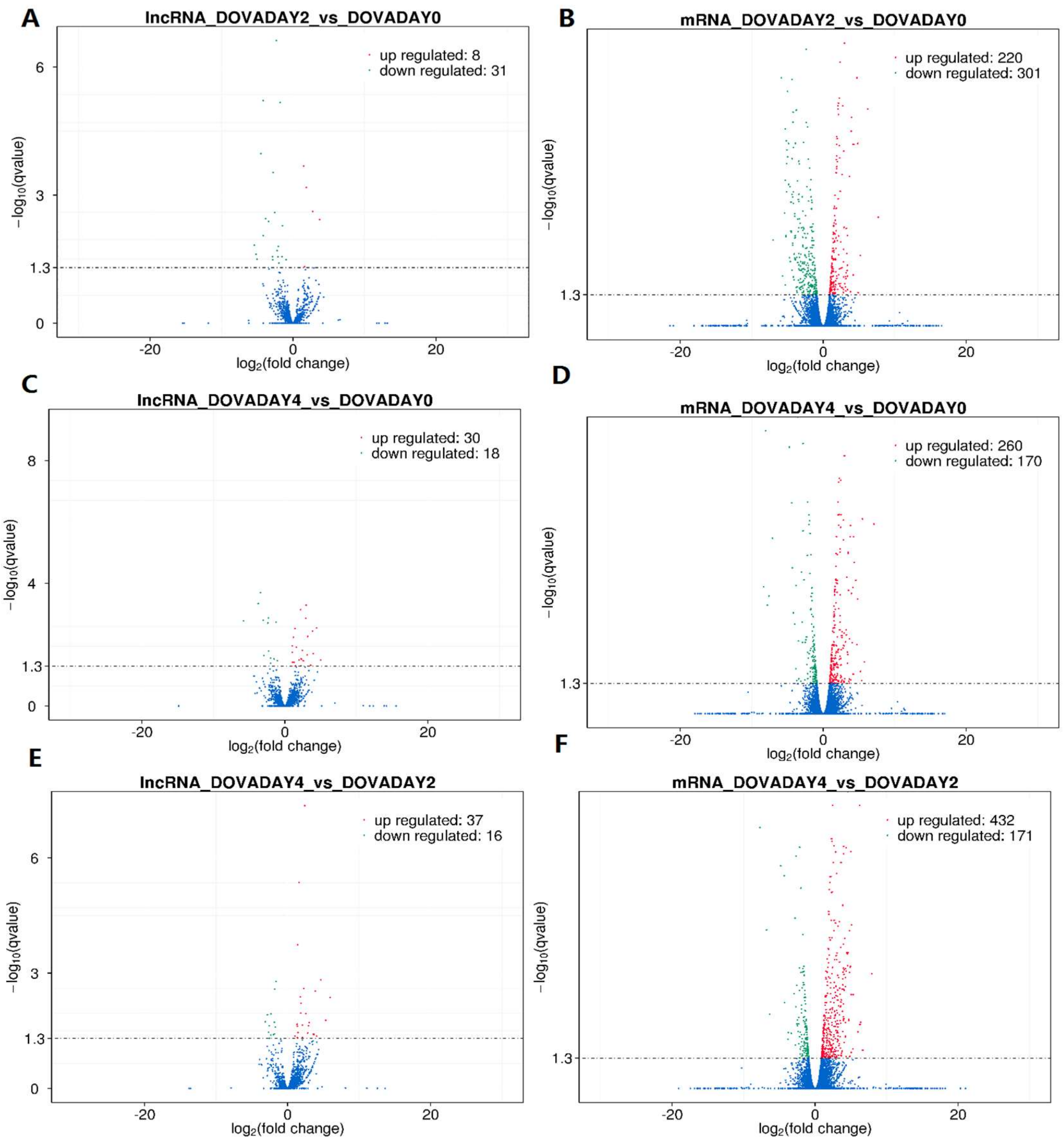

Supplementary Figure 3. 1694(140 lncRNAs and 1554 mRNAs) differentially expressed genes were obtained from pairwise comparison. A volcano plot of differentially expressed lncRNAs(A) and mRNAs(B) between DOVADAY2 and DOVADAY0, (C-D) between DOVADAY4 and DOVADAY0, and (E-F) between DOVADAY4 and DOVADAY2.

Note: the abscissa represents the transcripts expressed in different samples of multiple changes; the ordinate represents the statistical significance of the differences in the expression of transcripts; the red dots in the figure indicate the significant difference in expression of up - regulated transcripts; the blue dots indicate significant difference in expression of down - regulated transcripts.

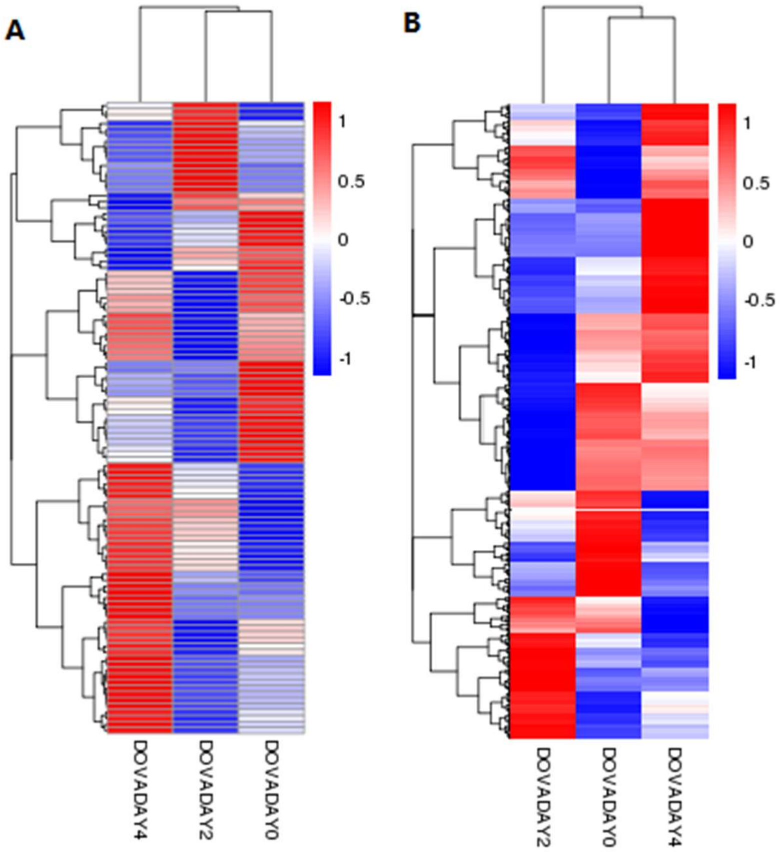

Supplementary Figure 4. Clustering thermogram of differential transcripts.

Note: (A): Differential expression of lncRNA clustered thermogram; (B): Differential expression of mRNA cluster thermogram; The overall FPKM hierarchical clustering map was clustered with log10 (FPKM + 1) values, red for high expression genes, and blue for low expression genes.

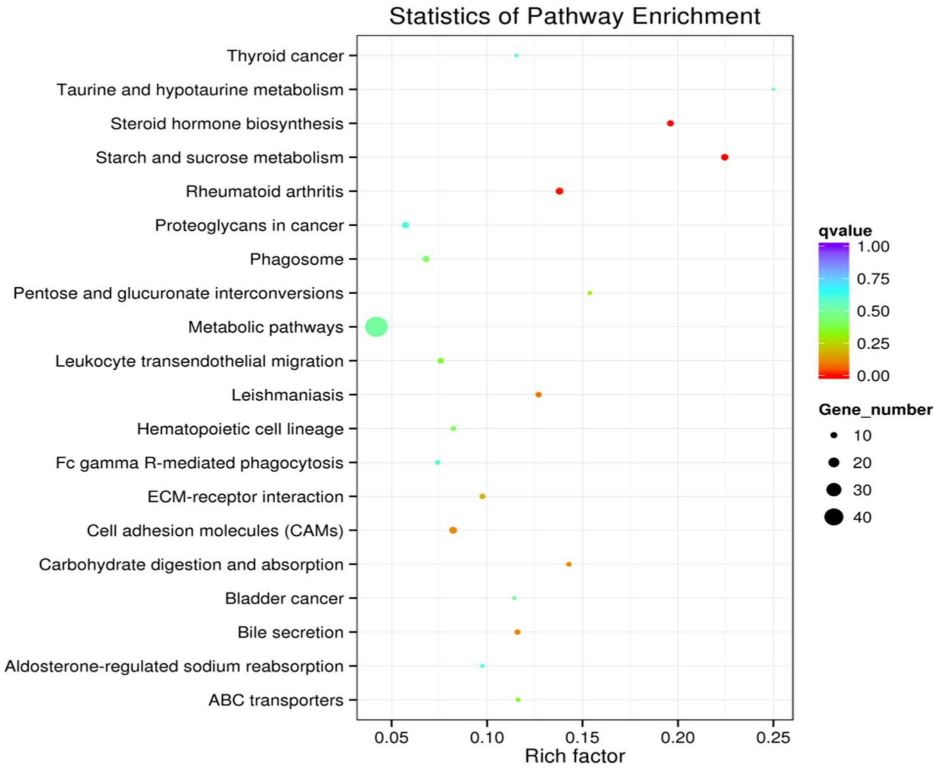

Supplementary Figure 5. KEGG enrichment scatter plot of differentially expressed mRNA in DOVADAY2 and DOVADAY0 group.

Note: rich factor refers to the ratio of the number of genes located in the pathway entry in the differentially expressed genes to the total number of genes in the annotated gene located in the pathway entry. The greater the Rich factor, the greater the degree of enrichment. Qvalue is the value of the pvalue after the multiple hypothesis test is corrected. The value range of qvalue is [0,1], the closer to zero, the more significant the enrichment is. We selected the most significant 20 study entries enriched in the map (the same as below).

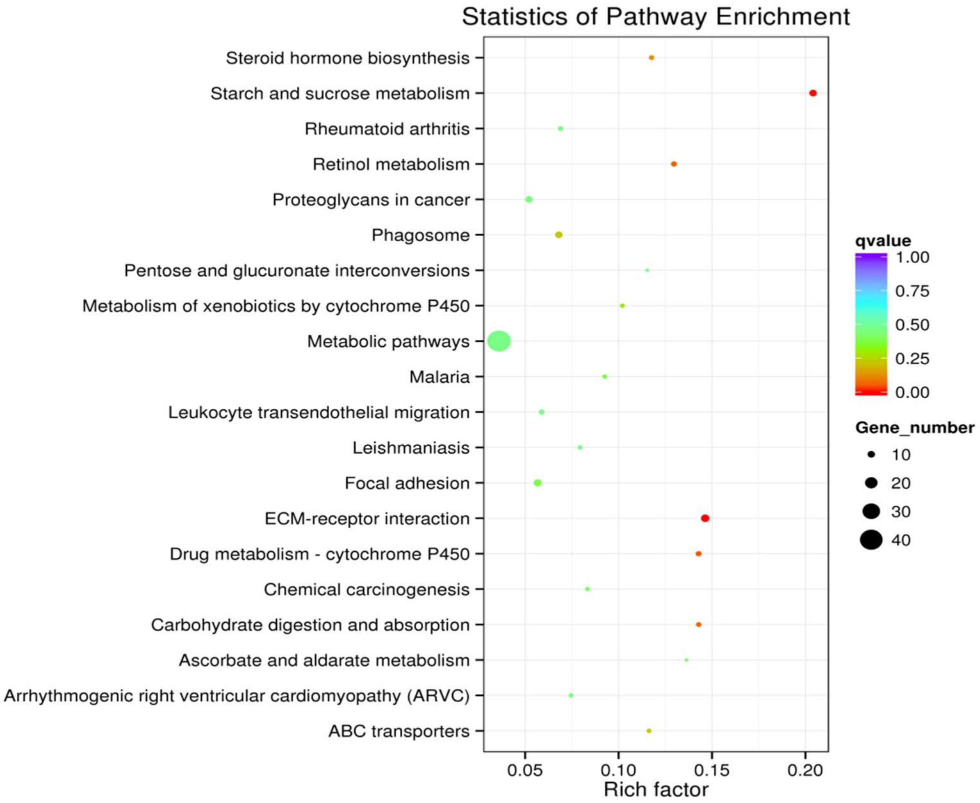

Supplementary Figure 6. KEGG enrichment scatter plot of differentially expressed mRNA in DOVADAY4 and DOVADAY0 group.

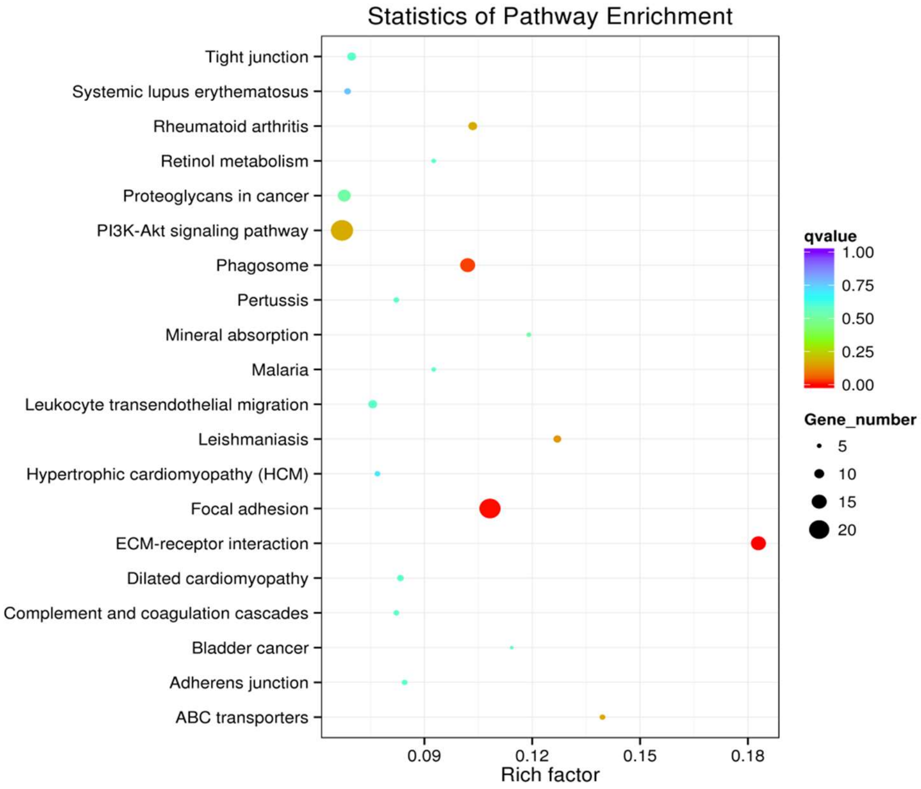

Supplementary Figure 7. KEGG enrichment scatter plot of differentially expressed mRNA in DOVADAY3 and DOVADAY4 group.
